# Effect of Fiber Content on Mechanical Properties of Fiber-Reinforced CGF All-Solid-Waste Binder-Solidified Soil

**DOI:** 10.3390/ma17020388

**Published:** 2024-01-12

**Authors:** Xinyi Qiu, Junjie Yang, Yalei Wu, Lijun Yan, Qiang Liu

**Affiliations:** 1 College of Environmental Science and Engineering, Ocean University of China, Qingdao 266100, China; qxy839540904@163.com (X.Q.); jjyang@ouc.edu.cn (J.Y.); yanlijun313@126.com (L.Y.); oucliuq@163.com (Q.L.); 2 The Key Laboratory of Marine Environment and Ecology of the Ministry of Education, Ocean University of China, Qingdao 266100, China

**Keywords:** fiber content, alkali-activated binder, tensile strength, fracture energy, reinforcement mechanism, tensile–compression ratio

## Abstract

In order to realize the resource utilization of solid waste and improve the tensile strength and toughness of soil, CCR-GGBS-FA all-solid-waste binder (CGF) composed of general industrial solid waste calcium carbide residue (CCR), ground granulated blast furnace slag (GGBS) and fly ash (FA) was used instead of cement and combined with polypropylene fiber to strengthen the silty soil taken from Dongying City, China. An unconfined compressive strength test (UCS test) and a uniaxial tensile test (UT test) were carried out on 10 groups of samples with five different fiber contents to uncover the effect of fiber content on tensile and compressive properties, and the reinforcement mechanism was studied using a scanning electron microscopy (SEM) test. The test results show that the unconfined compressive strength, the uniaxial tensile strength, the deformation modulus, the tensile modulus, the fracture energy and the residual strength of fiber-reinforced CGF-solidified soil are significantly improved compared with nonfiber-solidified soil. The compressive strength and the tensile strength of polypropylene-fiber-reinforced CGF-solidified soil reach the maximum value when the fiber content is 0.25%, as the unconfined compressive strength and the tensile strength are 3985.7 kPa and 905.9 kPa, respectively, which are 116.60% and 186.16% higher than those of nonfiber-solidified soil, respectively. The macro–micro tests identify that the hydration products generated by CGF improve the compactness through gelling and filling in solidified soil, and the fiber enhances the resistance to deformation by bridging and forming a three-dimensional network structure. The addition of fiber effectively improves the toughness and stiffness of solidified soil and makes the failure mode of CGF-solidified soil transition from typical brittle failure to plastic failure. The research results can provide a theoretical basis for the application of fiber-reinforced CGF-solidified soil in practical engineering.

## 1. Introduction

Tensile failure is one of the basic states of soil [1], as it is difficult for soil to resist tensile forces; under the action of external forces, soil structure is easily destroyed, leading to various engineering and geological problems. For instance, problems have been posed in the following areas: electric towers, due to the tension of the wire caused by tower foundation soil pile cracking; water conservation projects, with embankment slopes occurring under mixing piles due to bending or stretching and cracking, leading to destruction; and tunnels, with soil around the holes experiencing tensile stress, leading to tension cracks, etc. [2,3,4,5]. Therefore, it is of significant engineering importance to properly improve soil to effectively enhance its tensile strength.

Currently, soil improvement methods can be divided into three categories: chemical reinforcement, physical reinforcement and biological reinforcement [6]. Cement–soil is the commonly used chemical reinforcement method; its compressive strength has been greatly improved in comparison with soil, but it remains a relatively brittle material. Under brittle damage or tension cracking, the strength of cement–soil is reduced abruptly, and it is generally difficult to meet the deformation and stability requirements of engineering under complex stress conditions [7,8,9]. In order to increase the tensile strength and improve the plasticity of cement–soil, a composite reinforcement method combining chemical reinforcement and physical reinforcement by incorporating fibers has emerged in recent years [10,11]. In general, fibers have the advantages of high tensile strength and good dispersion, and they form an isotropic, high-tensile-strength composite with cement–soil [12,13,14,15,16]. The strength of fiber-reinforced, solidified soil is much greater than that of pure fiber–soil and solidified soil without fibers [17,18,19]. For example, the incorporation of PET fiber can effectively improve the tensile strength and durability of cement-based composite materials by providing corrosion resistance [20].

The soil type, binder type, curing condition, fiber type and content affect the strength of fiber-reinforced, solidified soil [21]. The combination of polypropylene fiber and fly ash can effectively increase the peak strain of cement–soil and maintain a certain residual stress after failure. Polypropylene fiber enhances the deformation resistance of fiber–fly ash cement–soil through anchorage and bridging, and improves its failure mode [3,22]. The addition of the plant fiber can effectively improve the tensile properties of cement-based materials, including the fracture energy and the tensile modulus, as well as increase the ductility of cement-based materials [23]. Simultaneously, the fiber enhances the tensile strength of solidified soil significantly more than the compressive strength [24]. As such, there is a good linear relationship between the tensile strength and compressive strength of fiber-reinforced solidified soil, i.e., the ratio of tensile and compressive strength is 0.105 [25]. In addition, the fiber content determines the strength variation in the fiber-reinforced, solidified soil to some extent. There is an optimum fiber content, where the strength increases with a fiber content below the optimum fiber content but decreases above the optimum fiber content [26]. The optimum fiber content is related to the material to be solidified and the fiber type [17,27,28,29,30]. On the other hand, the interaction between fibers and soil has a significant effect on the reinforcement mechanism of fiber-reinforced, solidified soil. The reinforcing effect of fibers mainly depends on the friction and adhesion of the fiber–soil, and the effect is influenced by soil conditions and interfacial contact conditions [25]. In cement–soil, the reinforcing effect of fibers and the cementing effect of cement hydration products on strength enhancement are not just a simple superposition, but enhance the strength of cement–soil through a complex coupling effect [31].

At present, most of the research on fiber-reinforced, solidified soil selects traditional binders such as cement and lime, and the use of all-solid-waste binders to replace traditional binders can alleviate the resource and energy consumption, ecological damage and environmental pollution brought about by the production process of traditional binders. Studies on fiber-reinforced, solidified soil mostly focus on sand and clay, and there are limited studies on silty soil. The tensile modulus, fracture energy and tensile–compression ratio of fiber-reinforced, solidified soil are also important indicators for characterizing tensile strength, and there remains a lack of research in this area.

In this paper, polypropylene fibers were selected as the reinforcing material, and an all-solid-waste binder (abbreviated as CGF) with calcium carbide residue (CCR), ground granulated blast furnace slag (GGBS) and fly ash (FA) as components was used to solidify the silt. The unconfined compressive strength test and the uniaxial tensile test were used to investigate the effect of fiber content on the mechanical properties of fiber-reinforced, solidified soil and the relationship between the tensile modulus and the deformation modulus, as well as between the tensile strength and the compressive strength. The results of this research can provide a theoretical basis for the application of fiber-reinforced, solid waste-based binder-solidified soil in practical engineering.

## 2. Materials and Methods

### 2.1. Materials

#### 2.1.1. Test Soil

**Figure 1:**
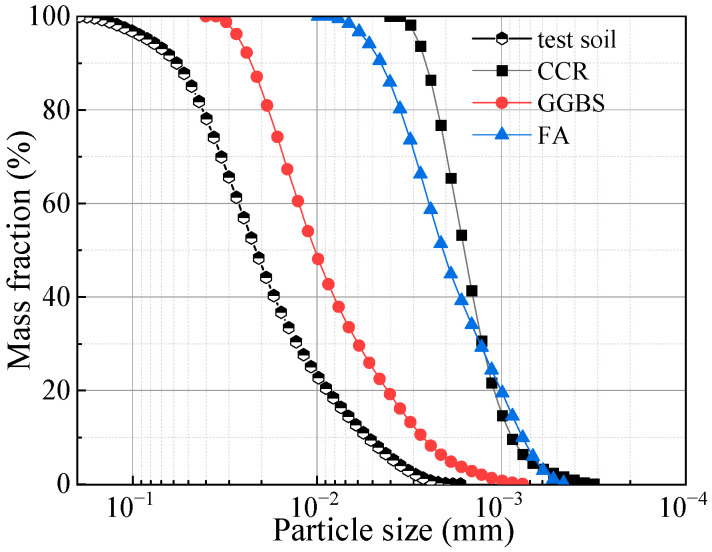
Particle size distribution curves of each component of binder and test soil.

The soil used in this study was taken from the subsurface soil (30–50 cm) of the first-class terrace near the estuary of the Yellow River in Dongying City, Shandong Province, China. The soil was dried for 12 h, crushed and sieved through a 0.075-mm sieve as the test soil. The basic physical properties of the test soil were tested according to the Standard for Geotechnical Test Methods [32]. The basic physical properties of the test soil are shown in Table 1; the cumulative curve of particle size gradation is shown in Figure 1, and the contents of clay and silt particles are 9.2% and 82.8%, respectively.

#### 2.1.2. CGF Binder and Polypropylene Fiber 

**Figure 2:**
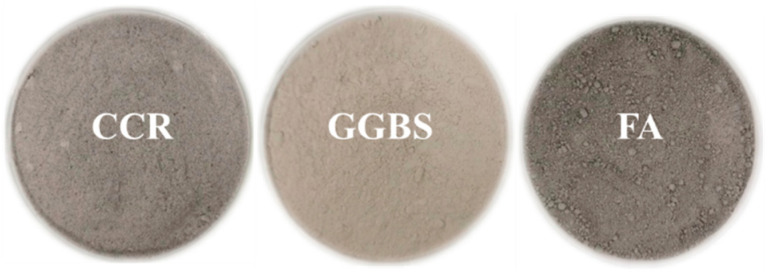
CGF binder’s components.

The CGF binder used in the test is an all-solid-waste, alkali-activated binder, which was prepared using raw calcium carbide residue (CCR), ground granulated blast furnace slag (GGBS) and fly ash (FA), according to a mass ratio of 4:4:2 (Figure 2) [33,34]. CCR is a by-product of acetylene obtained from calcium carbide hydrolysis, and its pH value exceeds 12.0, providing an alkaline environment as an alkali activator. GGBS is the waste residue produced in the iron-making process, and FA is fly ash collected by bag dust collectors in coal-fired power plants. The strength of CGF-solidified soils is 1.38–2.30 times higher than that of cement-solidified soil; however, the carbon emissions per unit strength of cement-solidified soils are 71.43–125.02 times higher than those of CGF-solidified soils, and the costs per unit strength are 3.53–5.88 times higher than those of CGF-solidified soils [32]. The chemical compositions of the components of the CGF binder are shown in Table 2, and the particle size distribution curves are shown in Figure 1.

**Figure 3:**
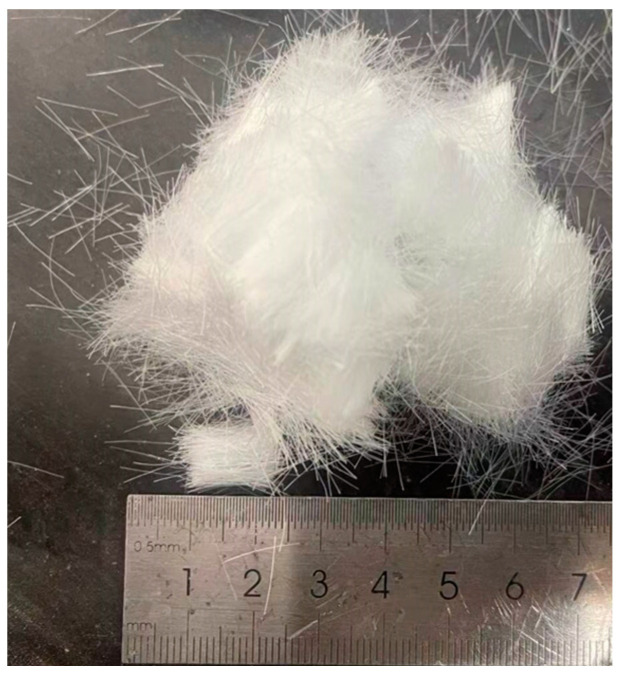
Polypropylene fiber for testing.

The reinforcing material selected for the test was polypropylene fiber with a length of 12 mm (Figure 3), and its physical and mechanical properties are shown in Table 3.

### 2.2. Testing Equipment

#### 2.2.1. Uniaxial Tensile Test Equipment

**Figure 4:**
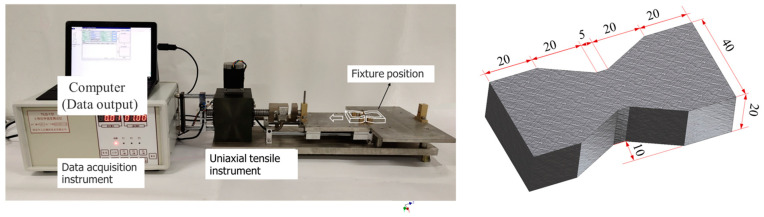
Uniaxial tensile test equipment and specimen size (unit: mm).

The uniaxial tensile test equipment is shown in Figure 4, including the uniaxial tensile platform, the data collector and the computer (data output). The loading mode was equal to the strain control, and the loading rate was set to 0.5 mm/min. The sample is an “hourglass” sample with a thickness of 20 mm. In order to limit the tensile failure location to a small range, the tensile section was set at 5 mm to alleviate the influence of stress concentration, and the guide was smooth with low friction [1].

#### 2.2.2. Unconfined Compressive Strength Test Equipment

**Figure 5:**
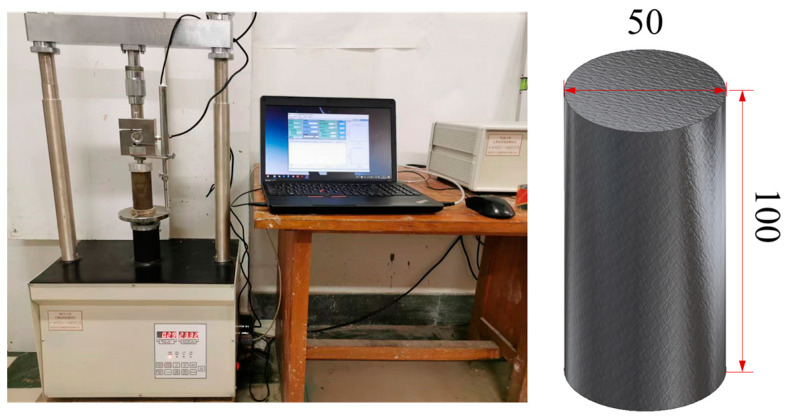
Unconfined compressive strength test equipment and specimen size (unit: mm).

The WCY-1 unconfined compressive strength test equipment is shown in Figure 5, used according to the Standard for Geotechnical Test Methods [32]. This device can apply a load to the sample in a controlled manner. The loading rate was set to 1 mm/min, and the maximum measuring range is 30 kN. The specimens are cylindrical, with a diameter of 50 mm and a height of 100 mm.

### 2.3. Testing Program

This paper focuses on the effect of fiber content on the tensile strength properties of fiber-reinforced CGF-solidified soil. For this purpose, the experimental program was designed as shown in Table 4. Fiber content is the mass ratio of fiber to dry soil and binder content is the mass ratio of binder to dry soil.

### 2.4. Sample Preparation Method

**Figure 6:**
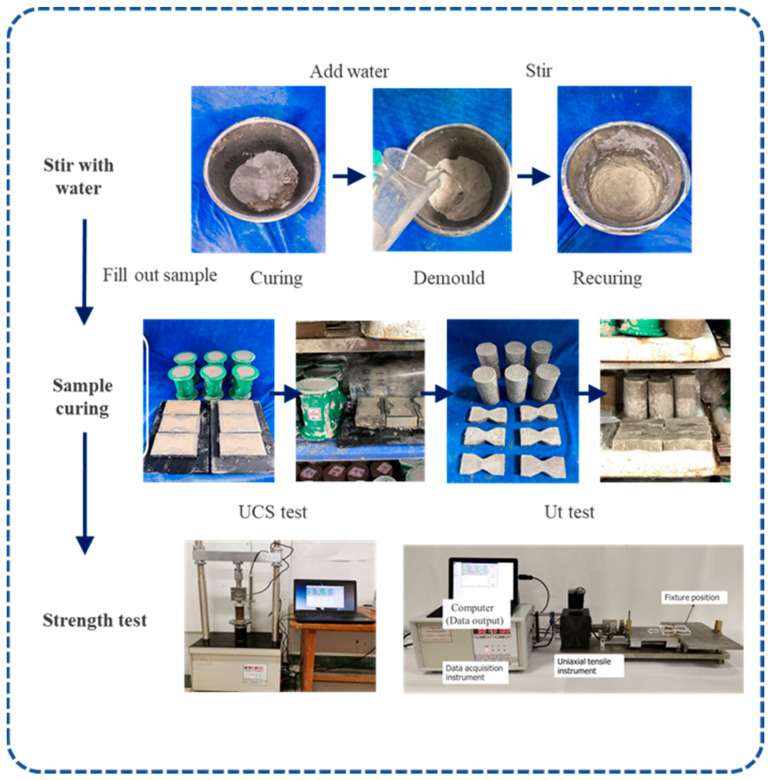
Sample preparation and testing process.

The binder was mixed with fiber and test soil to form a dry mixture, and water was added to the dry mixture to form a fiber-reinforced CGF-solidified soil mixture after mixing. The uniaxial tensile specimens were filled in a single layer, and the unconfined compressive specimens were filled in three layers. After filling the sample, the sample and the mold were placed into a curing chamber for standard curing (temperature 20 ± 2 °C, humidity ≥ 95%), and the mold was removed after 24 h. The demolded sample was placed in a sealed bag, and standard maintenance was continued until the set curing age, and then a uniaxial tensile test and an unconfined compressive test were carried out. After the strength test, the sample was broken, and a fresh section was selected for SEM testing. The sample preparation and testing process are shown in Figure 6.

## 3. Results and Discussion

### 3.1. Deformation Characteristics

#### 3.1.1. Stress–Strain Relationship and Failure Mode

**Figure 7:**
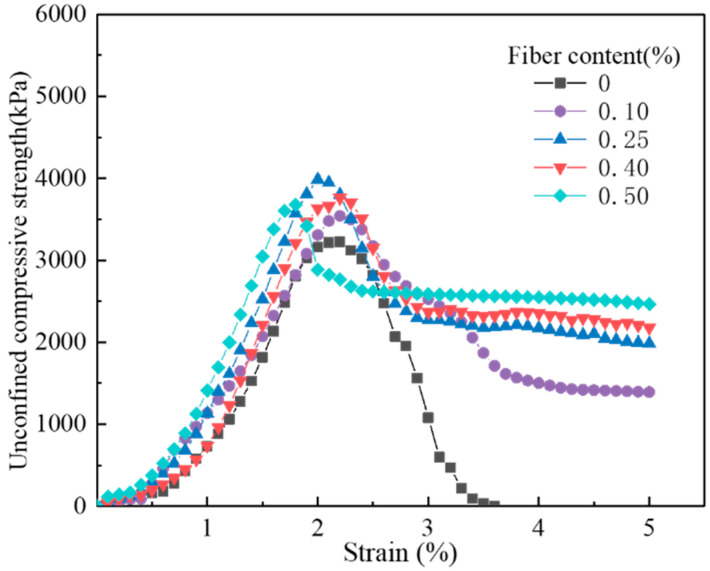
Unconfined compressive strength curve of fiber-reinforced, solidified soil.

Figure 7 shows the stress–strain curves of the fiber-reinforced, CGF-solidified soils with different fiber contents obtained from the unconfined compressive strength tests. The stress–strain curves of the solidified soils with and without fibers both showed peak stress. The peak stresses of the fiber-reinforced, CGF-solidified soils were all higher than those of the solidified soils without fibers, and the peak stresses of the solidified soils were the highest at a fiber content of 0.25%.

**Figure 8:**
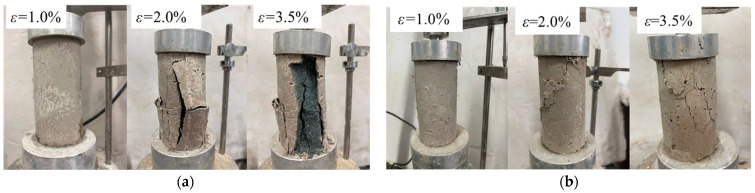
Failure morphology of unconfined compressive specimens at each stage: (**a**) fiber content: 0; (**b**) fiber content: 0.5%.

As can be seen in Figure 7, the stress–strain of the solidified soil increases linearly before reaching its peak stress. After the peak stress, the stress of the solidified soil without fiber dropped abruptly, the cracks induced by the damage extended in the load direction (Figure 8a), and the stress curve declined rapidly with the strain; at the same time, the specimens were seriously damaged and presented typical brittle failure characteristics. However, the stress of the fiber-reinforced CGF-solidified soil decreased slowly and maintained a certain residual stress, and the stress–strain curves show a strain-softening trend. Meanwhile, cracks appeared on the surface of the specimens and gradually lengthened and developed with the increase in stress, and the integrity of the specimen was still high under the adhesive effect of the fibers (Figure 8b); this represents plastic failure damage. In addition, the residual stress increased with the increase in fiber content. The above results indicate that fibers can effectively improve the toughness of solidified soil, and the failure mode of specimens gradually transitions from brittle damage to plastic failure [35]. The addition of fiber can increase the peak stress of solidified soil and retain some residual stress after failure, which is in line with fiber-reinforced cement soils [13,22,36].

**Figure 9:**
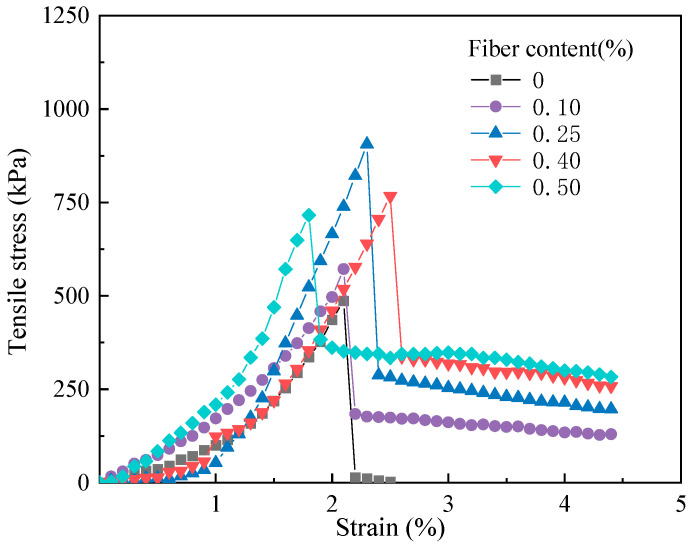
Tensile stress–strain curve of fiber-reinforced, solidified soil.

Figure 9 shows the tensile stress–strain curves of fiber-reinforced CGF-solidified soils with different fiber contents obtained from uniaxial tensile tests. The stress–strain curves of solidified soil with and without fibers both show peak stress. The peak tensile stresses of the fiber-reinforced CGF-solidified soils were all greater than those of the solidified soil without fibers, and the peak tensile stresses were the greatest at 0.25% fiber content.

As can be seen in Figure 9, the stress of the solidified soils with and without fibers showed a linear growth trend with the increase in strain before reaching the peak stress. The tensile stress of the solidified soil without fibers reaches its peak stress and then decreases sharply to zero, presenting a typical brittle failure. Therefore, the tensile stress of the fiber-reinforced CGF-solidified soil decreases slowly with the increase in strain, and there exists a longer strain-softening stage after dropping to approximately 20~40% of the peak stress, which is obviously different from the stress trend of the solidified soil without fibers. This indicates that the incorporation of fiber not only effectively enhanced the peak tensile stress of the solidified soil, but also improved its toughness.

**Figure 10:**
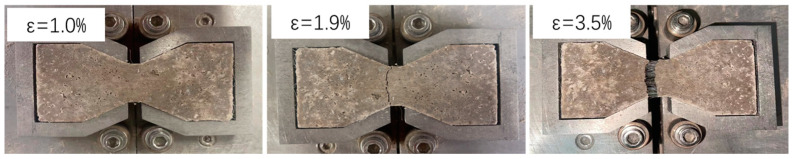
Failure morphology of uniaxial tensile specimen at each stage (fiber content 0.5%).

As the strain increases, the fiber-reinforced, CGF-solidified soil specimen produces cracks, and the tensile stress exceeds the maximum tensile stress produced by the solidified fiber-CGF hydration product-soil particle interface. As the tensile stress exceeds the pullout resistance of the fiber, tiny cracks appear perpendicular to the tensile direction in the tensile section, resulting in tensile damage. The cracks continue to expand and extend with the increase in strain, and the fibers play the role of a “Bridge”, which is responsible for transferring and dispersing tensile stress; therefore, the specimen began to retain a certain residual stress [18]. The residual stress increases with increasing fiber content and decreases slowly with increasing strain, and the fibers break and the specimen is completely destroyed when the tensile stress exceeds the tensile strength of the fibers (Figure 10). The strength of the solidified soil without fibers was completely destroyed after the peak tensile stress, with no residual stress.

**Figure 11:**
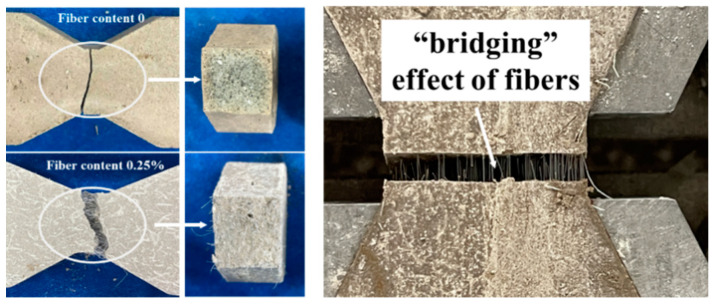
Tensile failure surface of fiber-reinforced solidified soil.

The tensile damage on the surface of the solidified soil with and without fibers (0.25% fiber content) is shown in Figure 11. The damage locations in the solidified soil were confined to a 5-mm-long neck stretch in the middle of the specimen, with the damage surface nearly perpendicular to the direction of stretching. The damage surface of the solidified soil without fibers is basically flat, which is typical brittle damage. Meanwhile, on the tensile damage surface of the fiber-reinforced, solidified soil at a fiber content of 0.25%, the fibers were partially pulled out and broken under the action of tensile stress, and some solidified soil particles adhered to the surface of the exposed fibers. The fibers play the role of a “bridge” across the fracture surface in the pulling process [16] to hinder further development of cracks and then improve the toughness of the specimens. The bridging effect of fiber in the process of CGF-solidified soil and the phenomenon of “cracking and ceasing” after fracture are consistent with the phenomenon of fiber acting on cement-solidified soil [13].

#### 3.1.2. Determination Method of Strength, Residual Strength and Modulus

**Figure 12:**
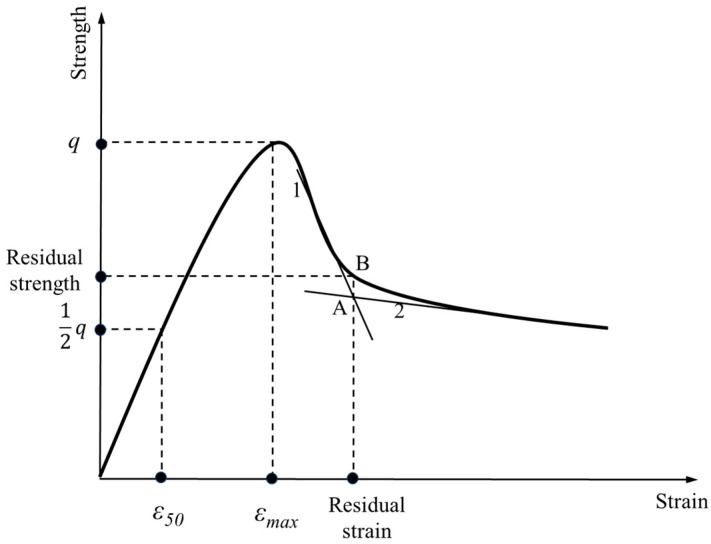
Definition diagram of indicators of strength and deformation characteristics.

As shown in Figure 12, the peak stress is taken as the strength, unconfined compressive strength is obtained from the unconfined compressive strength test (referred to as compressive strength, q*_u_*) and uniaxial tensile strength is obtained from the uniaxial tensile test (referred to as tensile strength, q*_t_*) [1]. The strain corresponding to the strength is the destructive strain (ε*_max_*). Take the stress corresponding to the point where the degree of reduction starts to slow down after the peak stress as the residual strength, i.e., set the intersection point of straight line 1, the section of sudden drop in stress after the peak, and straight line 2, the section of the stress–strain-stabilizing section, to be A; the transverse coordinate of the vertical line passing through point A and point B of the intersection point on the stress–strain curve to be the residual strain; and the longitudinal coordinate to be the residual strength.

The ratio of one-half strength to the corresponding strain is defined as the modulus. The deformation modulus (Equation (1)) [33] and the tensile modulus (Equation (2)) [1] are calculated as follows:

(1) $$E_{50u}=\frac{\frac{1}{2}q_{u}}{\varepsilon_{50u}}$$

where $E_{50u}$ is the deformation modulus, MPa; $q_{u}$ is the compressive strength, MPa; and $\varepsilon_{50u}$ is the strain corresponding to one half of the compressive strength, %.

(2) $$E_{50t}=\frac{\frac{1}{2}q_{t}}{\varepsilon_{50t}}$$

where $E_{50t}$ is the tensile modulus, MPa; $q_{t}$ is the tensile strength, MPa; and $\varepsilon_{50t}$ is the strain corresponding to one half of the tensile strength, %.

#### 3.1.3. Tensile Modulus and Deformation Modulus

The modulus indicates the stiffness of the soil and is an important physical quantity to describe the deformation characteristics of the soil [1]. The larger the tensile modulus E*_50t_*, the stronger the soil resistance to tensile deformation. Similarly, the larger the deformation modulus E*_50u_*, the stronger the soil resistance to compressive deformation [33,34].

**Figure 13:**
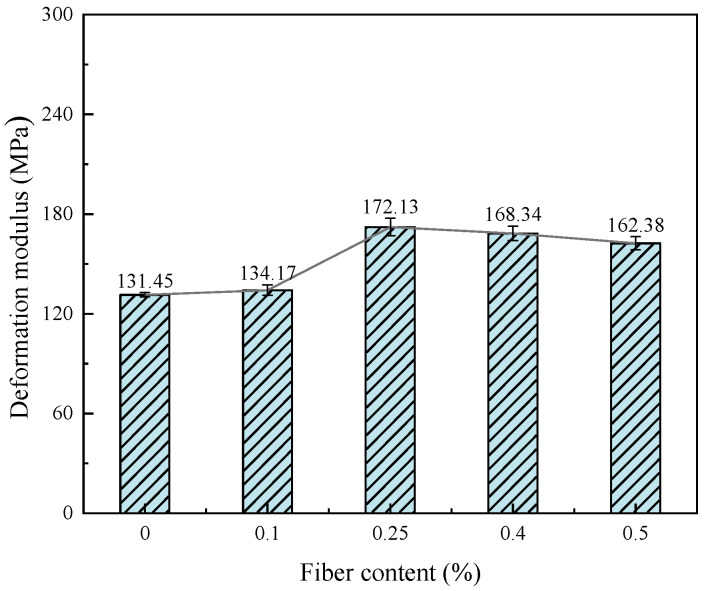
Relationship between deformation modulus and fiber content of fiber-reinforced, solidified soil.

**Figure 14:**
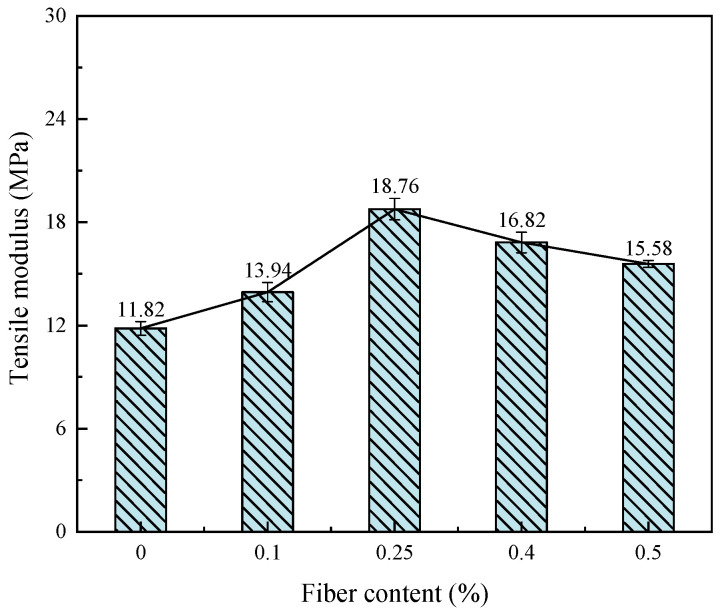
Relationship between tensile modulus and fiber content of fiber-reinforced, solidified soil.

Figure 13 and Figure 14 show the relationship between the deformation modulus, tensile modulus and fiber content of fiber-reinforced CGF-solidified soil, respectively. The addition of fiber increases the deformation modulus of solidified soil by 2.1–30.1% and the tensile modulus by 12.9–58.7%. The deformation modulus and tensile modulus both increased and then decreased with the increase in fiber content, reaching their peak value when the fiber content was 0.25%. When the fiber content was greater than 0.25%, the deformation modulus and tensile modulus showed a decreasing trend. The above phenomenon demonstrated that the low fiber content (less than 0.25%) is less distributed in the solidified soil, which is related to the limited contact area with the soil particles and the small interface friction. In this case, the fiber mainly plays the role of pillar reinforcement in the soil, which has poor resistance to deformation. When the fiber content is too high (greater than 0.25%), the excessive fiber congregates in the solidified soil, which tends to form a network of overhead fibers and does not come into contact with the soil particles, forming a weak stress zone and reducing friction. Macroscopically, the strength is reduced, and the resistance to deformation is reduced. However, the strengths of the fiber-reinforced CGF-solidified soils were still larger than those of the solidified soils without fibers. It is further shown that the incorporation of fiber can effectively improve the ability of solidified soil to resist deformation, and the effect of fiber on the resistance to tensile deformation is better than that of compressive deformation.

**Figure 15:**
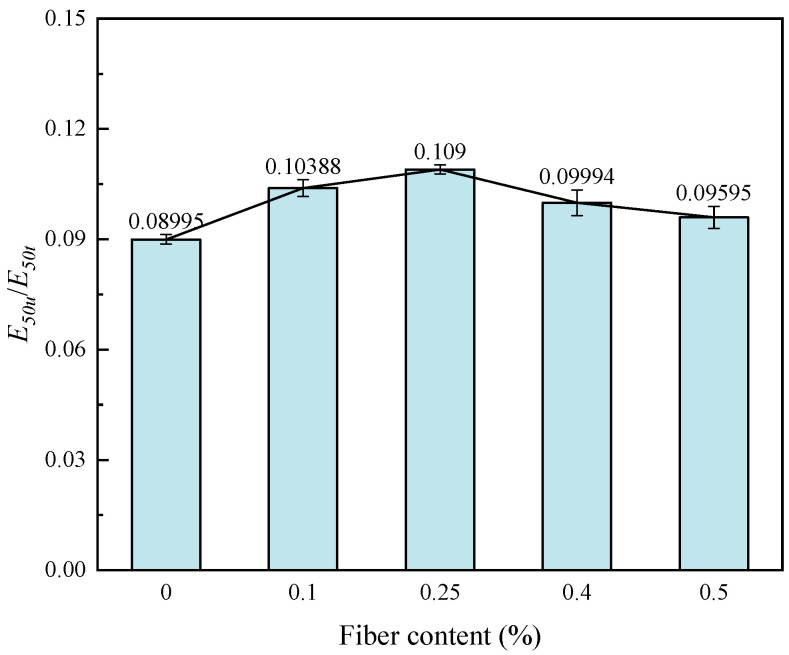
Relationship between *E*_50_/*E*_50*u*_ and fiber content of fiber-reinforced, solidified soil.

The ratio of tensile modulus to compressive modulus is called the modulus tensile–compression ratio, or tensile–compression ratio for short, which can reflect the ability of fiber-reinforced solidified soil to resist deformation under external forces. The higher the tensile–compression ratio, the stronger the resistance to deformation. The tensile–compression ratio shows a trend of increasing and then slowly decreasing with the increase in fiber content (Figure 15). This indicates that there exists an optimal fiber content (0.25%), which gives the fiber-reinforced CGF-solidified soil the strongest ability to resist deformation under tensile stress or external load. Nevertheless, the ability of fiber-reinforced CGF-solidified soils to resist deformation becomes weaker beyond the optimal fiber content.

#### 3.1.4. Fracture Energy

Fracture energy is the energy consumed on the fracture surface during the whole process, from the beginning of stretching to destruction, expressed in terms of the work exerted by the tension per unit of tension on the surface. Generally speaking, the larger the fracture energy, the stronger the material toughness. Fracture energy is an indicator for evaluating the toughness effect of fiber-reinforced, solidified soil [18,37] and is calculated using Equation (3).

(3) $$G_{F}=l\int_{0}^{\varepsilon_{t\max}}\sigma d\varepsilon$$

where $G_{F}$ is the fracture energy, kJ/m^2^; *l* is the specimen tensile section length, and the test *l* = 0.005 m; $\varepsilon_{t\max}$ is the destructive strain; σ is the stress, kPa; and ε is the strain.

**Figure 16:**
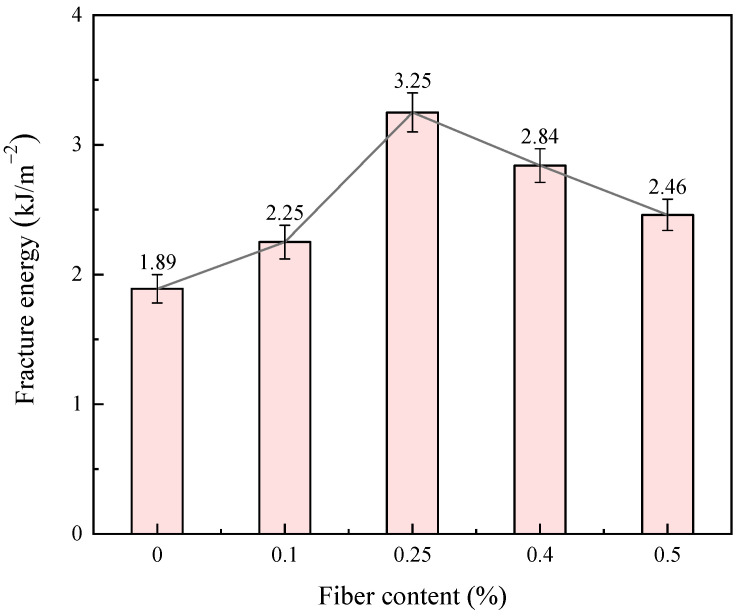
Relationship between fracture energy and fiber content of fiber-reinforced, CGF-solidified soil.

Figure 16 shows the relationship between fracture energy and fiber content. The fracture energy of the fiber-reinforced, CGF-solidified soil increased by 130.1~171.7% compared with the solidified soil without fibers. With the increase in fiber content, the tensile fracture energy of the sample first increases and then decreases, and the tensile fracture energy reaches its peak at 0.25% fiber content. This is due to the fact that fiber can increase the failure strain and peak tensile stress of the sample through friction and bonding, thus improving the tensile fracture of the sample; this indicates that fiber incorporation is conducive to enhancing the toughness of the sample. When the fiber content is 0.25%, the failure strain and failure stress are greater than those of the other four amounts of fiber content, and the energy consumed on the corresponding fracture plane is also the highest.

### 3.2. Strength Characteristics

#### 3.2.1. Influence of Fiber Content on Unconfined Compressive Strength

**Figure 17:**
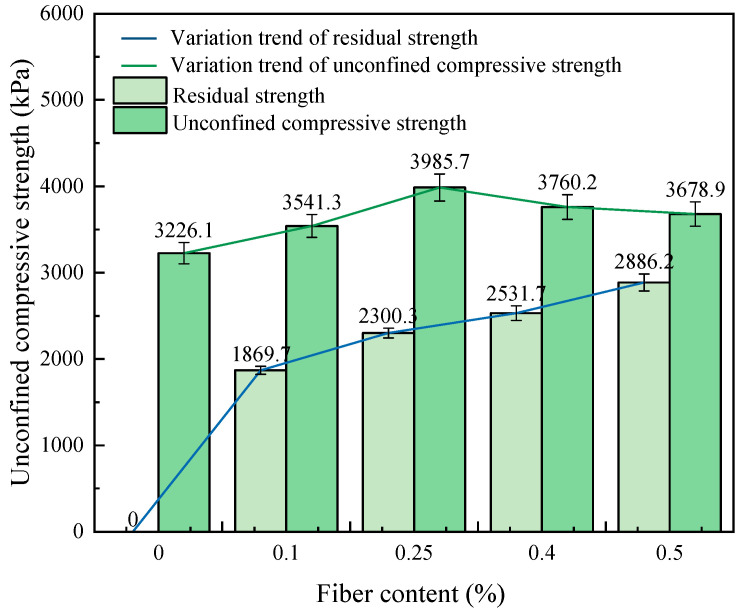
Relationship between UCS, residual strength and fiber content.

Figure 17 shows the relationship between compressive strength and fiber content. The compressive strength increases first and then decreases with the increase in fiber content. When the fiber content is 0.25%, the compressive strength reaches 3985.7 kPa. Compared with solidified soil without fibers, the compressive strength of solidified soil with fiber content of 0.1%, 0.25%, 0.4% and 0.5% increased by 109.8%, 123.6%, 116.6% and 114.0%, respectively. It can be seen that adding the appropriate amount of fiber to solidified soil can improve the compressive strength of the soil, and there is an optimal fiber content (0.25%). This is attributed to the fact that, as the fiber content is too low, the fiber is scattered in the solidified soil, and the reinforcement effect on the soil is only one-dimensional single fiber reinforcement. As the fiber content increased, the fiber formed a fiber network in the soil, and the structural strength and unconfined compressive strength of the soil increased significantly. When the fiber content is too high, there will be an uneven distribution phenomenon, which affects the overall durability of the soil and fiber combined force [28,38].

Fiber-reinforced, CGF-solidified soil retains a certain amount of residual strength after damage occurs, and the residual strength increases with increasing fiber content. The residual stresses of 0.1%, 0.25%, 0.4% and 0.5% were 184.1 kPa, 288 kPa, 337.1 kPa and 382.2 kPa, respectively. This is mainly due to the fact that the fibers on the fracture surface have the effect of bearing and dispersing certain stresses after damage to the specimen, and this effect is related to the number of fibers on the fracture surface. Therefore, unlike the unconfined compressive strength, the residual strength of fiber-reinforced, CGF-solidified soil did not reach its maximum at 0.25% of the fiber content, but was positively correlated with the fiber content.

#### 3.2.2. Influence of Fiber Content on Uniaxial Tensile Strength

**Figure 18:**
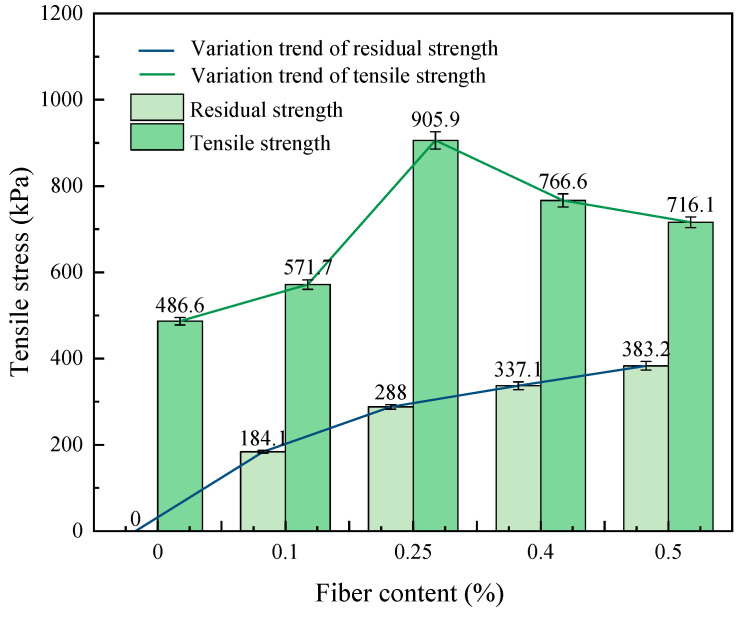
The relationship between tensile strength, residual strength and fiber content.

Figure 18 shows the relationship between the tensile strength of the fiber-reinforced CGF-solidified soil and the fiber content. The tensile strength of fiber-reinforced CGF-solidified soil increases and then decreases with the increase in fiber content. At fiber contents between 0 and 0.25%, the tensile strength increases with the increase in fiber content. Compared to the solidified soil without fibers, the tensile strength reaches its peak at 0.25% fiber content, 905.9 kPa, and increases by 186.16%. After exceeding 0.25% fiber content, the tensile strength remains higher than that of the solidified soil without fibers. Similar to the optimum fiber content for unconfined compressive strength, the fiber content also has an optimal fiber content to reach the peak uniaxial tensile strength, and this pattern is the same as the findings of other scholars [38,39]. The tensile residual strength of the fiber-reinforced CGF-solidified soil showed a positive correlation with fiber content and the same pattern as the compressive residual strength.

The above result is due to the low fiber content (0–0.10%), where the fiber distribution in the soil is relatively dispersed, the contact area with the solidified soil particles is limited and the interface friction generated by the two is also small, making it difficult to form a network space structure. The main role of the fiber in the solidified soil is to provide a one-dimensional reinforcement effect with low-strength macroscopic mechanical properties. As the fiber content increases (0.10–0.25%), the fiber forms a three-dimensional spatial grid structure in the solidified soil, which can transfer and disperse tensile stress [40]. The hydration products formed by CGF have high bonding strength, which can effectively improve the bonding force between the fiber and the soil; at the same time, these hydration products will wrap the fiber to improve the stiffness of the fiber. When the soil is subjected to tensile stress, the fibers play one-dimensional and three-dimensional roles at the same time, limiting the relative sliding of soil particles through friction and bonding forces, and the macroperformance is significantly improved in terms of integrity and strength. When the fiber content is too high (0.25–0.50%), excessive fiber is easily wound into a group in the stirring process, and the distribution is uneven, forming a weak force area; thus, the number of effective fibers bearing the tension is reduced, which affects the durability of the fiber and the solidified soil under stress, and the macro performance is that the strength of the sample is reduced.

### 3.3. Relationship between Tensile–Compression Ratio of Strength and Fiber Content

**Figure 19:**
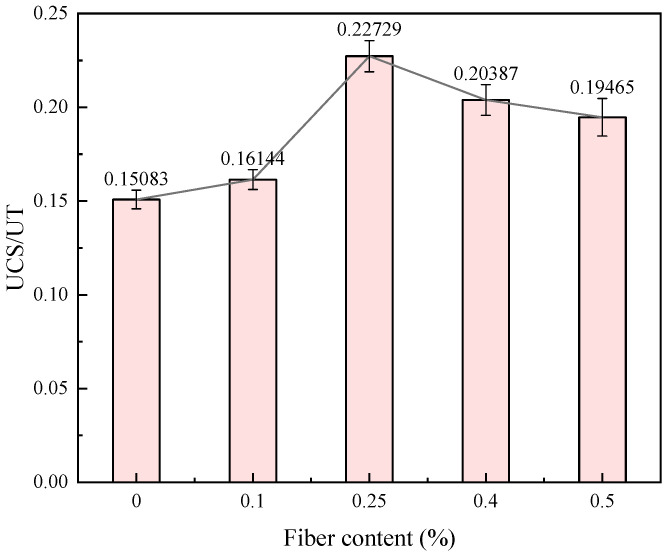
The relationship between UCS/UT and fiber content.

The tensile–compression strength ratio is the ratio of tensile strength to unconfined compressive strength, or simply the tensile–compression ratio, which can be used to assess a material’s brittle characteristics [41]. The relationship between the tensile–compression ratio and fiber content of the fiber-reinforced CGF-solidified soil is shown in Figure 19. With the increase in fiber content, the tensile–compression ratio first increases and then decreases. The tensile–compression ratio of the solidified soil without fibers is the smallest, which is 0.151. The maximum tensile–compression ratio of 0.227 occurs in the fiber-reinforced CGF-solidified soil at 0.25% fiber content. This indicates that the fiber content can significantly improve the toughness of solidified soil, and the optimal fiber content is 0.25%; when exceeding this fiber content, the toughness of solidified soil will grow slowly, but it is still greater than the toughness of solidified soil without fibers.

### 3.4. Micromechanisms

#### 3.4.1. Microscopic Morphology

**Figure 20:**
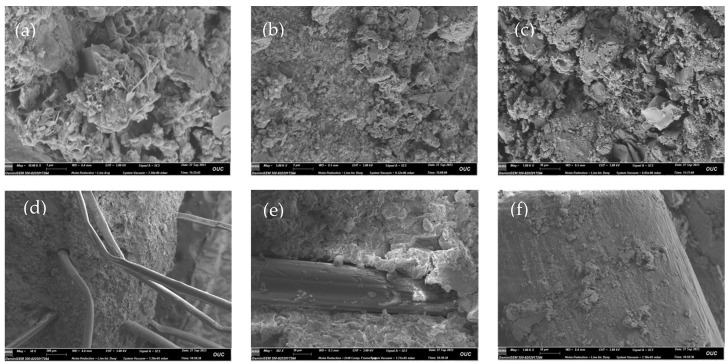
SEM diagram of fiber CGF-solidified soil: (**a**) ×10,000, (**b**) ×5000, (**c**) ×1000, (**d**) ×50, (**e**) ×200, (**f**) ×1000.

The results of the SEM test are shown in Figure 20. As seen in Figure 20a, the spaces between the soil particles are filled with needle-like, flocculent and blocky CGF hydration products. These hydration products cemented the soil particles and fibers into a whole, partly filled in the pores (Figure 20b,c) and partly attached to the fiber surface (Figure 20f).

After magnifying 50 times, it can be seen that the fibers are randomly distributed in the solidified soil in a three-dimensional mesh structure, and some of the fibers are bent under the action of external force (Figure 20d); after magnifying 200 times, it can be observed that there exists an anchoring effect at the interface of the fibers and the CGF-solidified soil, so the fibers cannot be easily pulled and pulled out (Figure 20e); after magnifying 1000 times, it can be observed that the fibers’ surface is adhered to the CGF hydration products, making the originally smooth fibers rough and uneven (Figure 20f).

#### 3.4.2. Fiber–Binder Coupling Reinforcement Mechanism

**Figure 21:**
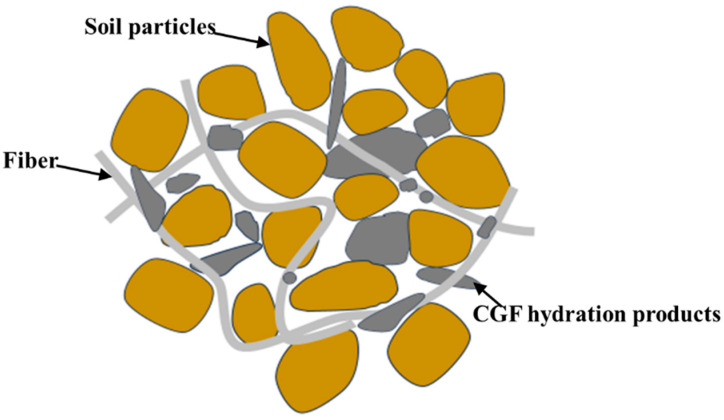
Interface diagram of fiber/CGF-solidified soil.

Combined with the results of the unconfined compressive strength, uniaxial tensile and microscopic tests, the fiber–binder coupling reinforcement mechanism was analyzed: the cementing and filling effect of CGF (solidification effect), the embedding and bridging effect of fibers (reinforcing effect) and the spatial reticulation structure are the main factors controlling the reinforcement effect. A mechanism diagram is shown in Figure 21.

1. Gelling and filling effect of CGF: In the alkaline environment formed by the dissolution of a large amount of OH^−^ from CCR in contact with water, GGBS and FA undergo the pozzolanic reaction to generate hydration products such as C-A-H, C-S-H and C-A-S-H [33,34], which can gel the soil particles and fibers, fill the pore spaces in the soil and increase the density of the soil, increasing the strength of solidified soil.
2. Embedding and bridging effect of fibers: The enhancement effect of fibers on the strength of solidified soil depends on the mechanical interaction between fibers and the interface of the solidified soil matrix. As the hydration products of CGF have strong gelling properties, they attached to the fiber surface to form a layer of hard shell, which effectively enhanced the stiffness of the fiber. At the same time, the crystals attached to the fiber surface and the hydration products inside the solidified soil combined with each other, resulting in the overall firm embedding of the fiber in the solidified soil. Due to the large difference in the modulus of elasticity between the fiber and the solidified soil, the difference in deformation between the two produces relative displacement; thus, the originally smooth fiber surface attaches to the solidified soil particles, resulting in its surface roughness and unevenness, which increases the occlusal force of the fiber–solidified soil interface and improves the ability of the fiber to bear tensile stress. Under the action of external forces, the interface forces of adhesion and friction can inhibit the fiber’s slip in the solidified soil, preventing the fiber from being pulled out. When the external force continues to increase, the soil body becomes damaged, and fiber plays a “bridging” role through the fracture surface to inhibit the expansion of the damaged surface; when the external force exceeds the force in the fiber-solidified soil interface or the fiber’s own strength, then the fiber will be pulled out of the soil. When the external force exceeds the force at the fiber-solidified soil interface or the strength of the fiber itself, the fiber on the fracture surface will be pulled out or broken, and the reinforcing effect of the fiber will be completely lost [42,43,44].
3. Spatial network structure: When the optimal fiber content (0.25%) is used in the CGF-solidified soil, the fibers cross and overlap each other inside the soil, presenting a randomly distributed mesh structure. When one or more bundles of fibers in the mesh structure are subjected to relative movement with the solidified soil, the fibers overlapping with them will transfer and disperse the stresses in different directions and improve the overall strength of the solidified soil [45]. However, when the fiber content is too high, the excess fiber will be unevenly distributed in the solidified soil and knotted into a ball, resulting in the existence of “weak areas” in the soil but also damage to the structure of the soil, thus reducing its strength [46].

## 4. Conclusions

This paper proposes the use of polypropylene-fiber-reinforced CGF all-solid-waste binder (abbreviated as CGF)-solidified soil modified by carrying out unconfined compressive strength and uniaxial tensile tests, and the effects of different fiber contents on the deformation characteristics and strength characteristics of the CGF-solidified soil were comparatively analyzed. The micro-mechanism and reinforcement mechanism under fiber-CGF coupling were also analyzed with SEM results. The following main conclusions were obtained:

- Compared with the nonfiber-solidified soil, the unconfined compressive strength, uniaxial tensile strength, deformation modulus, tensile modulus, fracture energy and residual strength of the fiber-reinforced CGF-solidified soil are significantly improved. Compared with the solidified soil without fiber, the fracture energy of the fiber-reinforced CGF-solidified soil can be increased by 130.1–171.7%, the deformation modulus can be increased by 102.1–130.1% and the tensile modulus can be increased by 112.9–158.7%. The increased amplitudes of the tensile strength and tensile modulus are greater than those of the compressive strength and deformation modulus, respectively. The addition of fiber makes the failure mode of the CGF-solidified soil transition from typical brittle failure to plastic failure.
- The compressive strength and tensile strength of the polypropylene-fiber-reinforced CGF-solidified soil increased and then decreased with the increase in fiber content, and both reached their maximum values when the fiber content was 0.25%. The tensile strength is 905.9 kPa, which is 186.6% higher than that of the solidified soil without fiber, and the compressive strength is 3985.7 kPa, which is 116.6% higher than that of the solidified soil without fiber. However, both the compressive residual strength and tensile residual strength increased continuously with the increase in fiber content, and there was no optimal content.
- The modulus tensile–compression ratio and strength tensile–compression ratio of the fiber-reinforced CGF-solidified soil both increased and then decreased with the increase in fiber content, reaching the maximum at 0.25% fiber content. The modulus tensile–compression ratio is 0.109 and the strength tensile–compression ratio is 0.227. The incorporation of fiber effectively improved the toughness and stiffness of the solid soil.
- The fiber-CGF coupling effect is mainly embodied by two processes: first, the gelling and filling effect of the hydration products generated by CGF, enhancing the compactness of the soil body and then enhancing the strength of the soil body; second, the one-dimensional embedding and bridging effect of the fibers and the three-dimensional spatial mesh structure, resulting in a combination of friction and adhesion, preventing the development of cracks and enhancing the ability of the soil body to resist deformation.

## Figures and Tables

**Table 1:** Basic physical properties of test soil.

Particle Density (*G_s_*)	Plastic Limit (%)	Liquid Limit (%)	Plasticity Index (*I*_P_)
2.68	26.5	29.7	3.2

**Table 2:** Chemical compositions of components of CGF binder.

	Chemical Component	CaO	SiO_2_	Al_2_O_3_	MgO	Fe_2_O_3_	Na_2_O	K_2_O	SO_3_	P_2_O_5_
Component										
CCR		68.8	3.59	1.56	1.21	0.09	/	0.028	0.75	/
GGBS		41.17	29.47	13.61	8.04	0.425	0.676	0.354	4.90	0.03
FA		6.60	61.29	12.66	0.02	4.48	3.75	1.32	0.66	0.01

**Table 3:** Physical and mechanical parameters of polypropylene fiber.

Type	Tensile Strength (MPa)	Ultimate Elongation in Percent (%)	Elasticity Modulus (MPa)	Diameter (μm)	Length (mm)
Fasciculate monofilament	469	28.4	4236	32.7	12

**Table 4:** Testing program.

Test Soil	Water Content	Binder Type	Binder Content (%)	Fiber Content (%)	Curing Age (d)	Type of Test
Dongying silt	1.2*w_L_*	CGF	15	0, 0.1, 0.25, 0.4, 0.5	28	Uniaxial tensile test, inconfined compression test, SEM

Note: CGF is prepared using CCR, GGBS and FA according to the mass ratio of 4:4:2.

## Data Availability

Some or all of the data, models or codes that support the findings of this study are available from the corresponding author upon reasonable request.
